# Young children learn first impressions of faces through social referencing

**DOI:** 10.1038/s41598-021-94204-6

**Published:** 2021-07-20

**Authors:** Adam Eggleston, Elena Geangu, Steven P. Tipper, Richard Cook, Harriet Over

**Affiliations:** 1 Department of Psychology, University of York, York, YO10 5DD UK; 2 Department of Psychological Sciences, University of London, London, UK

**Keywords:** Psychology, Human behaviour

## Abstract

Previous research has demonstrated that the tendency to form first impressions from facial appearance emerges early in development. We examined whether social referencing is one route through which these consistent first impressions are acquired. In Study 1, we show that 5- to 7-year-old children are more likely to choose a target face previously associated with positive non-verbal signals as more trustworthy than a face previously associated with negative non-verbal signals. In Study 2, we show that children generalise this learning to novel faces who resemble those who have previously been the recipients of positive non-verbal behaviour. Taken together, these data show one means through which individuals within a community could acquire consistent, and potentially inaccurate, first impressions of others faces. In doing so, they highlight a route through which cultural transmission of first impressions can occur.

## Introduction

Humans spontaneously attribute a wide range of traits to strangers based on their facial features^1^. These first impressions include judgements about honesty, competence, intelligence, dominance, and likeability^1–4^. First impressions are thought to load on two principle dimensions—trustworthiness and dominance^1^. Developmental research has demonstrated that the tendency to form impressions from faces emerges early in development. By the age of 3, children make explicit judgements about how ‘nice’ and ‘strong’ a person is after viewing images of their face^5,6^. These first impressions exert a measurable influence over behaviour. Ewing and colleagues have shown that children are more generous towards individuals who appear trustworthy^7^. Interestingly, research with adults has shown that while some first impressions contain a kernel of truth^8^, others bare little or no resemblance to the actual character traits of the individuals being judged^9^.

A key priority for developmental research is to understand the mechanism, or mechanisms, by which first impressions of faces are acquired^10,11^. One view is that at least some first impressions are the product of an innately specified mechanism to distinguish between potential social partners^12–14^. According to this view, determining who among many social partners was a potential collaborator or leader was so crucial to our evolutionary ancestors that natural selection favoured individuals who were predisposed to make these judgments^12,13^. In contrast, Trait Inference Mapping (TIM) offers a learning account in which all mappings between ‘face space’ and ‘trait space’ are acquired as a result of experience^11^. Consistent with this, research has shown that adults quickly learn who is trustworthy from receiving small amounts of behavioural information about them^15^ and generalise this information to novel individuals who are somewhat similar in appearance^16^.

Proponents of both theoretical positions agree that at least some first impressions must be learned^11,17–23^. Evidence in favour of this claim comes from data showing that participants form first impressions from cultural cues that are the products of recent human history. For example, children and adults from Western cultures typically judge individuals who wear glasses to be more intelligent than individuals who do not wear glasses^24^. Other research has shown that there are systematic cultural differences in first impressions^10^ and that it is possible to modify pre-existing first impressions of faces with training^16,22^.

To date, relatively little research has directly investigated *how* face-trait learning takes place. One particular challenge is to explain how inaccurate first impressions can emerge through learning^25^. If individuals learned face-trait mappings through direct experience, then consistent first impressions would not emerge at a group level because people’s facial appearance rarely predicts their actual character traits^9^. TIM explains the prevalence of shared but inaccurate impressions through appealing to cultural learning^11,26^. One route through which this could happen is exposure to cultural products such as storybooks, films, advertising and propaganda that pair particular facial features with character traits^11^. For example, children’s animations pair the presence of physical beauty in protagonists with positive behaviours^27,28^. Through exposure to such systematic messages, many children in the same community may acquire similar face-trait mappings.

The authors of TIM also predicted that children within a community might learn common (but inaccurate) first impressions from their caregivers^11^. Sometimes, teaching could take the form of explicit instruction. For example, verbal warnings that children should avoid individuals with a particular physical appearance. Other forms of teaching might be more implicit. Specifically, children might learn first impressions partly through social referencing^11^. That is, by attending to the non-verbal responses of others^29^.

We know from previous research that adults’ non-verbal behaviour differs in systematic ways when interacting with individuals who differ in appearance. For example, Weisbuch and colleagues^30^ have shown that the non-verbal behaviour of Caucasian Americans in popular TV shows is less positive when interacting with African Americans than when interacting with other Caucasian Americans (see also: Castelli et al.^31^). Furthermore, both children and adults can acquire intergroup biases from observing the non-verbal behaviour of others^30,32^. Indeed research on social referencing suggests that even infants use the nonverbal reactions of others to decide who to approach and avoid^33^.

Here, we extend this research to the domain of first impressions of faces and investigate whether children use the non-verbal behaviour of others, specifically similar aged peers, to infer the trustworthiness of faces. We chose to investigate the influence of peers on first impressions as previous research has suggested that peers exert a consistent influence over children’s choices and preferences in social settings^34^. We test this question with 5- to 7-year-olds because we know that children in this age range form consistent first impressions on the basis of others’ appearance^5,7,35^ and engage in extensive social learning^36^.

### Study 1

The main aim of this study was to determine whether children use the non-verbal behaviour of others to make attributions of facial trustworthiness. We presented children with computerised displays in which target faces were paired with context faces that appeared either happy or afraid. We predicted that when children were asked “who do you think is nicer” that they would choose target faces associated with happy context faces significantly more than target faces paired with fearful faces. Following previous developmental research in this area, we used the term “nice” rather than “trustworthy” as the younger children in our sample may not understand the term “trustworthy”^35,37^. We also sought to test whether children generalise from these learned associations to similar looking but novel individuals. We predicted that children would show a preference for the composites constructed from targets previously associated with happy context faces.

## Method

### Pre-registration and open science

Both studies were pre-registered. The pre-registered details for Study 1 (https://aspredicted.org/blind.php?x=tu6ci3) and Study 2 (https://aspredicted.org/blind.php?x=45bc27) are available. The data is also available open access at Open Science Framework: (https://osf.io/eu4q8/?view_only=4c882227705549f0a547a58f35270c7b).

### Participants

The final sample consisted of 120 children, with equal numbers of 5-year-olds (20 boys M_age_ = 66 months, age range = 60 to 71 months), 6-year-olds (20 boys M_age_ = 76 months, age range = 72 to 82 months), and 7-year-olds (20 boys M_age_ = 90 months, age range = 84 to 95 months). An additional four children were tested but excluded from analysis in line with our pre-registered exclusion criteria (they required more than 4 prompts to look at the screen). Of the 120 participants included in the analysis, 106 were identified by their parents as White British, 1 as White European, 1 as White Irish, 2 as British/Indian, 5 as British/Pakistani, 1 as British/Bangladeshi, 1 as White/Black Caribbean, 1 as Asian Mixed, 1 as White and Black African and 1 as Mixed English/Arab. All children were recruited from a science museum in an urban centre and were tested on site the same day. Informed written consent was gathered from a parent of every child tested and assent was gained from each child. The procedure was approved by the University of York Department of Psychology’s Ethics Committee and all methods were performed in accordance with the committee’s guidelines.

### Materials

**Figure 1:**
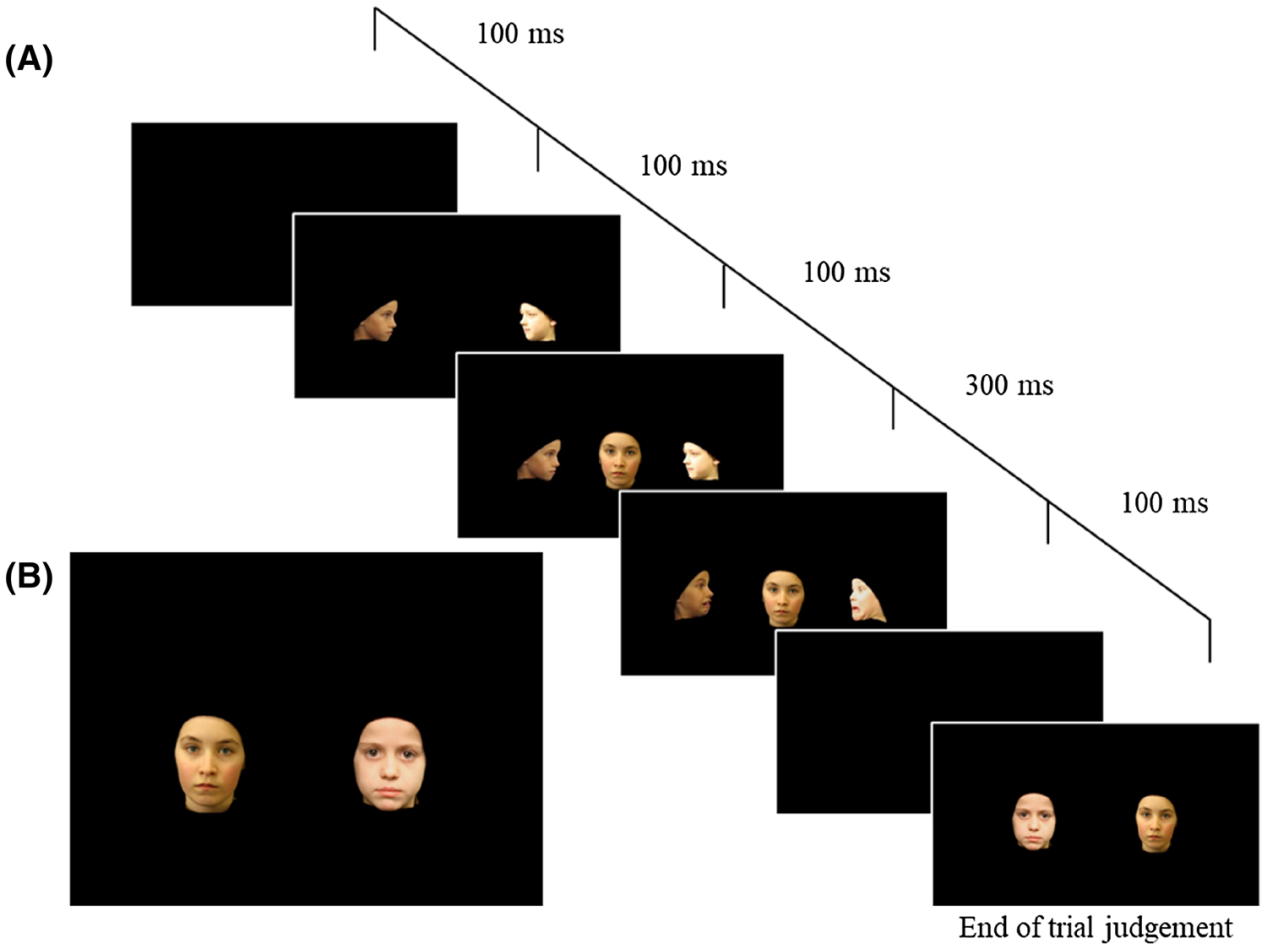
Stimuli in Study 1. (**a**) Schematic representation of frames from a learning event incorporating fearful context faces (**b**) Example test trial.

The stimuli were photographs of children’s faces taken from the Dartmouth Database of Children’s Faces^38^ and all faces used in the figures throughout are those for which Dalrymple and colleagues obtained assent from the child and written informed consent from the parent to both have their photographs distributed to other researchers and for the photographs to be used in scientific publications. This database was chosen as it provided high quality and constrained images of children’s faces featuring a range of expressions at different angles. The ages of the children depicted in the photographs ranged from 8 to 10 years, were all white, and contained an equal number of males and females. The background was removed from each image and faces were presented on a black background. Target faces were presented facing the camera and context faces were presented in profile and appeared to observe the target face (see Fig. 1a). In each of the 4 context pairs, one face was female and one was male.

It is possible that baseline levels of trustworthiness will affect learning and so we chose pairs of target faces that resembled each other as closely as possible in apparent trustworthiness. In order to achieve this, we ran a pre-test in which we asked 20 undergraduate participants to rate the children’s faces on a 100 point slider-scale ranging from ‘not at all nice/trustworthy’ to ‘extremely nice/trustworthy’. Target pairs were then matched based on gender and average nice/trustworthiness ratings. The relative trustworthiness of the 16 targets are available as Supplementary Table S1 online.

Stimuli for the two generalisation trials were created using WebMorph^39^ an online programme specifically created for image manipulation and transformation (see^40,41^ for a detailed description of the process). The generalisation stimuli are the result of averaging the 4 target faces previously paired with happy context faces and the 4 target faces previously paired with fearful context faces to create 4 composite images in total—two for the male target faces and two for the female target faces.

### Design

Participants completed 8 standard trials, each with a unique pair of faces, followed by a final generalisation test (a male test and female test). Within each standard trial, there were 8 learning events; 4 in which one of the target faces acquired positive valence through context faces moving from a neutral to happy expression, and 4 in which the other target face in the pair acquired negative valence through context faces moving from a neutral to fearful expression (Fig. 1a). The 8 learning events lasted 49 s in total. At the end of each trial, participants were shown the two target faces side-by-side (see Fig. 1b) and were asked a forced choice question: ‘Who do you think is nicer, this person or this person?’.

Half of trials included male target faces and half included female target faces. Half of trials begin with happy context faces and half begin with fearful context faces. In addition, the ‘trustworthy’ face appeared on the left in 50% of trials. The target faces associated with happy and fearful context faces were switched in two counterbalancing conditions, and within these counterbalancing conditions trials were presented in one of two possible orders. Overall, this resulted in four between subjects counterbalancing conditions to which participants were randomly assigned.

### Procedure

Participants were invited into the testing area and asked to sit at a small table in front of a laptop. After a brief warm up, the experimenter (E) conducted a comprehension test to assess children’s understanding of the term nice. To do this E presented a pair of male faces and said: “First I am going to tell you about two different people. This person shared a cookie with another person in their class and this person stole a cookie from another person in their class. Who do you think is nicer? This person or this person?” [Pointing to each picture in turn]. Children were corrected if they chose incorrectly and a note made of their decision.

The first of the experimental trials began with E saying: “Now I’m going to show you some more people, a bit like these, and it’s going to play a bit like a video. At the very end of the video I am going to ask you again who you think is the nicest, okay? Please try and look at the screen for the whole video.” E then started the presentation of the first trial. Following the onset of the two target faces (side-by-side), E asked, ‘Who do you think is nicer—this person or this person?’ [Pointing to each picture in turn]. The same procedure and wording was used for all remaining trials. To ensure children stay engaged with the task, after every two trials there was a short break where children were offered a sticker to add to their bookmark. The two generalisation trials followed immediately after the final test trial with no break in between. Children were presented with two more pairs of target faces (one male pair and one female pair) and were asked again ‘Who do you think is nicer—this person or this person?’ [Pointing to each picture in turn].

### Coding

Participants were given a score out of 8 for the number of times they chose the target faces that had been paired with happy context faces. For the generalisation trials, children were given one score for the female target pair and one score for the male target pair. 25% of the data were second coded by a rater who did not know the hypotheses of the study. There was perfect agreement between the two coder's judgements, κ = 1.

## Results

Following our pre-registered analysis plan, we conducted a one-sample *t*-test evaluating the number of times the children (*N* = 120) chose the target individuals associate with smiling context faces, against a chance level of 50% (i.e., a score of 4 out of 8). Overall children were more likely to pick the target associated with smiling faces (*M* = 5.25, *SD* = 1.84) above chance [*t*(119) = 7.43, *p* < 0.001, Cohen’s *d* = 0.68].

**Figure 2:**
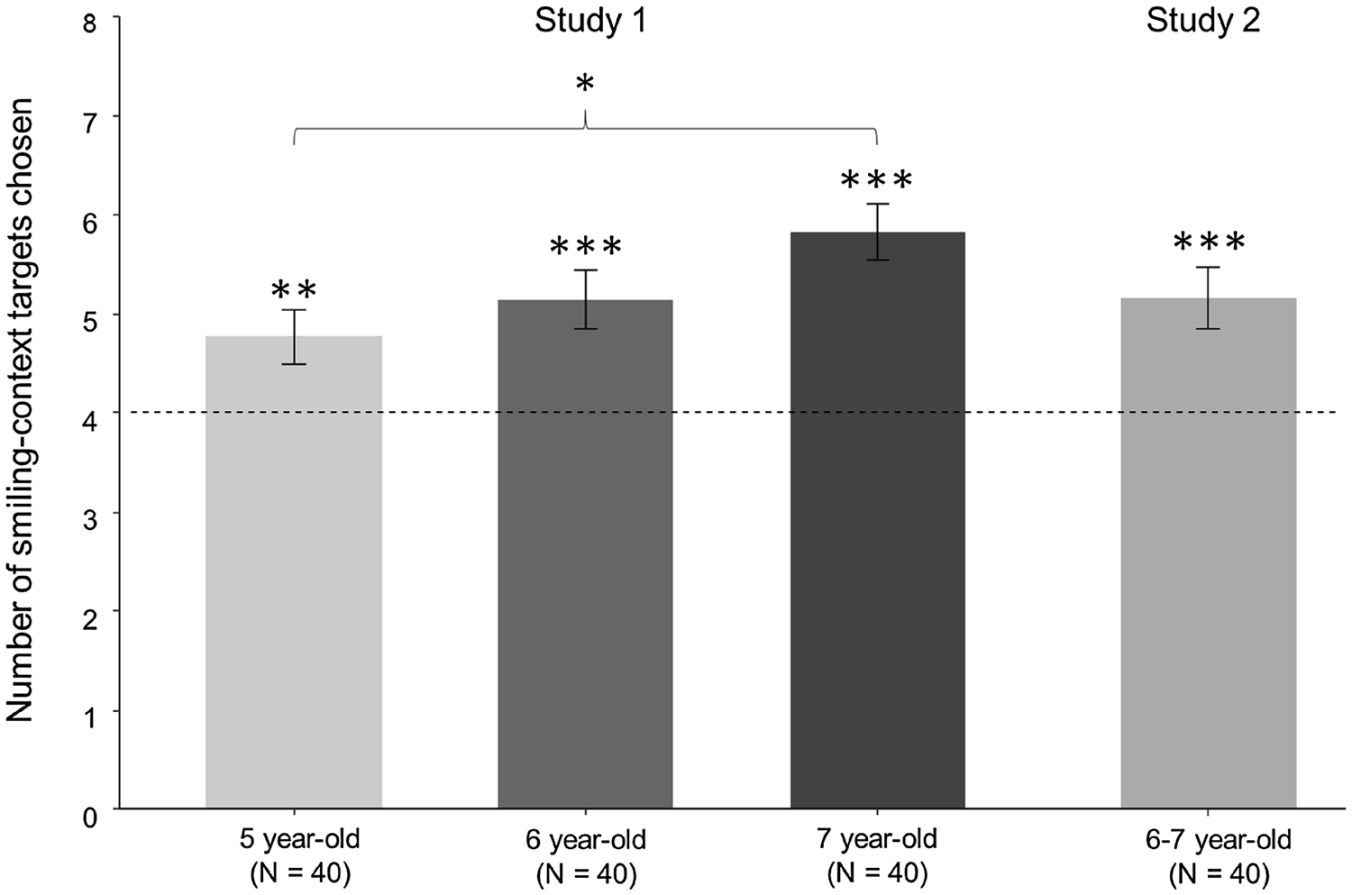
Results from Studies 1 and 2 by age group. Error bars represent ± SEM. Dashed line indicates chance performance. Asterisks represent a result significantly different from chance. *denotes *p* < .05, **denotes *p* < .01; ***denotes *p* < .001.

Again following our pre-registered plan, follow-up tests were conducted to analyse performance at each age group separately. One-sample *t*-tests revealed that 5-year-olds (*N* = 40) [*t*(39) = 2.83, *p* = 0.007, Cohen’s *d* = 0.45], 6-year-olds (*N* = 40) [*t*(39) = 3.82, *p* < 0.001, Cohen’s *d* = 0.61], and 7-year-olds (*N* = 40) [*t*(39) = 6.479, *p* < 0.001, Cohen’s *d* = 1.02] all chose the target associated with smiling faces above chance (see Fig. 2).

In order to compare performance between age groups we also performed a one-way ANOVA with age as a factor. A significant effect of age was found, *F*(2,117) = 3.47, *p* = 0.034, *η*^2^ = 0.06. Post hoc comparisons using Tukey HSD tests indicated only one significant comparison: 5-year-olds chose the target face associated with smiling context faces significantly less than 7-year-olds (*p* = 0.028). No other comparisons were statistically significant (all *p*’s > 0.221).

In addition to our pre-registered analysis we conducted an exploratory regression to investigate further the influence of age on task performance. We regressed age in months against the number of trials on which children chose the target faces associated with happy context faces. This analysis found a significant regression equation (*F*(1,118) = 6.80, *p* = 0.010), with an R^2^ of 0.055. The number of trials on which children chose the target associated with happy context faces increased by 0.53 for each month of age.

Performance on generalisation trials was assessed using separate Chi-squared tests for male and female trials. Overall, participants (*N* = 120) chose the morph of the male children shown in the happy context on 44.17% of trials. They chose the morph of the female children shown in the happy context on 53.33% of trials. The observed frequencies in the male [*X*_(1)_^2^ = 1.63, *p* = 0.201] and female [*X*_(1)_^2^ = 0.53, *p* = 0.465] generalisation tests did not differ significantly from chance. Further chi-squared tests revealed that this was true across all ages (all *p*’s > 0.114).

## Discussion

In Study 1 we showed that children aged 5–7 are able to use the non-verbal behaviour of similar aged peers to make inferences about how “nice” a target is. These results extend previous research on social referencing to the domain of faces and show that social referencing is one route through which children can learn first impressions of faces.

We did not find evidence that children generalise their learning to similar looking individuals. In order to measure generalisation, we used morphing to blend together the four target faces that had been paired with happy and fearful contexts, respectively. It was hoped that this approach would produce novel male and female identities that resembled those individuals encountered during the learning episodes. It is possible, however, that unique visual information associated with each target identity was lost. Consistent with this possibility, previous findings suggest that composite images constructed from relatively few faces start to approximate the “average” face of the population^42,43^.

### Study 2

In Study 2, we sought to further test the role of social referencing in the formation of first impressions from faces using a similar design. Similar to Study 1, there were 8 trials. Each trial was composed of 8 learning events; 4 in which one target face was seen in a smiling context, and 4 in which a second target face was presented in a fearful context. This time, however, the target faces presented at the end of each trial were morphs. Each target contained 70% of one of the two target identities and 30% of a novel identity. Thus, each target face was somewhat novel, but bore a strong resemblance to one of the trained faces. We predicted that morphs featuring target faces associated with happy context faces would be viewed as more trustworthy than morphs featuring target faces paired with fearful faces. We sought to conceptually replicate the results of Study 1 and, in doing so, test whether children would generalise from learned faces to similar looking faces.

### Participants

We chose to test a combined sample of 6- and 7-year-olds as children in these two age groups performed similarly in Study 1. We employed a sample size of 40 based on an a priori power analysis conducted using G*Power3^44^. Using the overall effect size from Study 1 (Cohen’s *d* = 0.68) results showed that a minimum sample size of 25 participants was required to achieve power of 0.95. The final sample for this study consisted of 40 children (20 boys M_age_ = 83 months, age range = 72 to 94 months). An additional child was tested but excluded from analysis due to interruption during testing. Of the 40 participants included in the analysis, 39 identified as White British and 1 as English/Greek Cypriot. All children were recruited from either a science museum in an urban centre or a primary school in Northern England. Informed written consent was gathered from a parent of every child tested and assent was gained from each child. The procedure was approved by the University of York Department of Psychology’s Ethics Committee and all methods were performed in accordance with the committee’s guidelines.

## Materials and design

The materials and design used in Study 2 were identical to that of Study 1 with the exception of the test trials. Test trials consisted of face morphs containing 70% (shape, colour, texture) of the target faces from the training trials and 30% from previously unseen faces. Members of each pair were morphed with the same previously unseen face using WebMorph^39^.

### Procedure

Study 2 followed the same procedure as Study 1 with two exceptions. There was no final “generalisation trial” after test trials had finished, and children were now prompted to look at the screen before each learning event to minimise the risk of exclusions. Coding was identical to that of Study 1.

### Coding

A rater naïve to the hypothesis of the study second coded 25% of the data. There was near perfect agreement between the two coder's judgements, κ = 0.957, *p* < 0.001. The one disagreement between the coders was resolved by discussion.

## Results

Following our pre-registered analysis plan, we conducted a one-sample *t*-test evaluating the number of times children chose the morph featuring the target previously associated with happy context faces, against a chance level of 50% (i.e., a score of 4 out of 8). Overall children chose the target associated with smiling context faces (*M* = 5.18, *SD* = 1.99) significantly more often than chance [*t*(39) = 3.74, *p* = 0.001, Cohen’s *d* = 0.59] (see Fig. 2). This replicates and extends the results of Study 1. Although we did not find evidence of generalisation in Study 1, with the more sensitive method adopted in Study 2, children did use social referencing to learn about the facial trustworthiness of individuals and generalised their social learning to very similar images.

### General discussion

We investigated how the non-verbal behaviour of others influences young children’s first impressions of faces. Study 1 showed that, at least from the age of five, children use the non-verbal appraisal of similar aged peers to infer the apparent trustworthiness of others faces. This replicates and extends previous research demonstrating that children are sensitive to the non-verbal behaviour of others, using it to inform their understanding of the social world^32,45^. Study 2 showed that children generalise their learning to novel individuals who clearly resemble individuals who have previously been the recipients of positive non-verbal behaviour^15,16,46^. This supports the claim that social referencing is one route through which children can learn to form spontaneous first impressions of others’ faces^11,26^.

These results accord with the broader literature on social learning from nonverbal cues. Previous research has shown that young children learn intergroup biases from observing others’ nonverbal responses^31,32,45^ and that they use the nonverbal behaviour of teachers to infer the intelligence of their peers^47^. We extend this important research to the domain of first impressions from faces and show that children use the non-verbal behaviour of peers to decide which faces appear trustworthy.

Previous research has noted an apparent paradox whereby first impressions are widely shared across individuals but contain, at most, a kernel of truth^48^. Our studies suggest that social referencing is one route through which consistent but inaccurate first impressions of faces could emerge^11^. Participants in the current studies received no direct evidence relating to each target’s trustworthiness. Rather, they learned about apparent trustworthiness through the nonverbal behaviour of others. While nonverbal behaviour of this sort may reflect veridical information about the targets, it can also reflect shared stereotypes^30^.

TIM predicts that face-trait mappings will gradually approach adult-like patterns and levels of consistency throughout development as children are exposed to more systematic messages about face-trait relationships^11^. Consistent with this view, Cogsdill and colleagues^6^ found that younger children (3- to 4-year-olds) make less consistent trait judgements than older children (7- to 10-years-old). Our finding that older children seem better able to learn about the traits of others through social referencing, reveals another interesting aspect of this developmental trajectory. Previous findings that older children exhibit stronger and more consistent trait inferences from faces may, in part, reflect older children’s greater exposure to correlated face-trait mappings. However, it may also reflect the fact that older children, perhaps due to more mature face processing abilities^49^ or better categorisation skills^50^, are better equipped to learn about their social world.

There is broad agreement in the field that at least some first impressions from faces must be the product of learning^19,51,52^. The work reported here builds on previous research on social referencing in other areas to demonstrate one route through this face-trait learning could occur. Importantly, however, these results do not rule out the possibility of an innate contribution to first impressions. It is possible that social learning of this type builds on an innate foundation of face-trait mappings in order to produce the consistent first impressions observed in adults^7^. It is also possible that first impressions differ in their origins and that some first impressions are more heavily reliant on social learning than others. Research in this area has shown that some first impressions are strongly influenced by the emotional expression of the target, whereas others appear to be based on the target’s facial features^53^. It may be that social learning plays a more important role in explaining the latter than the former.

An outstanding question is whether children could generalise to more distantly related individuals. In Study 2, targets closely resembled the faces used at training. Although previous research speaks to 6 and 7 year olds’ ability to discriminate between highly similar faces^54^ it is still possible that children perceived the faces used at test as the same identities as those used in training. In future research, it would be interesting to test whether children also generalise their learning from non-verbal responses to faces that more distantly resemble the faces used at training^46,55^. It would also be interesting for future research to investigate the independent effects of observing happy and fearful non-verbal displays, perhaps through incorporation of a baseline condition in which the target faces appear without context faces. Another limitation of our research is that the stimuli were entirely composed of white children. An important avenue for future research is to assess how face-trait learning generalises to more diverse stimuli and how intergroup biases may interact with face-trait learning. By focusing the study of first impressions on the developmental processes by which they are acquired, these studies suggest a number of other important avenues for future research. It would be interesting to investigate the range and limits of the inferences children make following exposure using a range of dependent variable and to explore the extent to which first impressions can be modified by altering the available cultural input. This endeavour may ultimately have applied implications as it suggests that the content of storybooks, films and TV could be manipulated in order to alter children’s first impressions^11^.

Overall, these findings highlight the important role of cultural learning in explaining how children learn first impressions and, in doing so, help explain the apparent paradox by which first impressions are widely shared between members of a community but often inaccurate^9,56^.

## Supplementary Information

Supplementary Information.

## Data Availability

The data that support the findings of this study are openly available at Open Science Frameworkc https://osf.io/eu4q8/?view_only=4c882227705549f0a547a58f35270c7b.

## References

[1] Oosterhof NN, Todorov A (2008). The functional basis of face evaluation. Proc. Natl. Acad. Sci..

[2] Sutherland CAM (2013). Social inferences from faces: Ambient images generate a three-dimensional model. Cognition.

[3] Todorov A, Olivola CY, Dotsch R, Mende-Siedlecki P (2015). Social attributions from faces: determinants, consequences, accuracy, and functional significance. Annu. Rev. Psychol..

[4] Zebrowitz LA, Montepare JM (2008). Social psychological face perception: why appearance matters. Soc. Personal. Psychol. Compass.

[5] Charlesworth TES, Hudson STJ, Cogsdill EJ, Spelke ES, Banaji MR (2019). Children use targets’ facial appearance to guide and predict social behavior. Dev. Psychol..

[6] Cogsdill EJ, Todorov AT, Spelke ES, Banaji MR (2014). Inferring character from faces: a developmental study. Psychol. Sci..

[7] Ewing L, Sutherland CAM, Willis ML (2019). Children show adult-like facial appearance biases when trusting others. Dev. Psychol..

[8] Bonnefon JF, Hopfensitz A, De Neys W (2015). Face-ism and kernels of truth in facial inferences. Trends Cogn. Sci..

[9] Dilger A, Muller J, Muller M (2017). Is Trustworthiness written on the face?. SSRN Electron. J..

[10] Over H, Eggleston A, Cook R (2020). Ritual and the origins of first impressions. Philos. Trans. R. Soc. B Biol. Sci..

[11] Over H, Cook R (2018). Where do spontaneous first impressions of faces come from?. Cognition.

[12] Schaller, M. Evolutionary bases of first impressions. in *First Impressions* 15–34 (2008).

[13] Zebrowitz, L. A. & Zhang, Y. Origins of impression formation in animal and infant face perception. In *The handbook of social neuroscience* (Oxford University Press, 2011).

[14] Zebrowitz LA (2004). The origin of first impressions. J. Cult. Evol. Psychol..

[15] Verosky SC, Todorov A (2010). Generalization of affective learning about faces to perceptually similar faces. Psychol. Sci..

[16] Verosky SC, Todorov A (2013). When physical similarity matters: Mechanisms underlying affective learning generalization to the evaluation of novel faces. J. Exp. Soc. Psychol..

[17] Hackel LM, Doll BB, Amodio DM (2015). Instrumental learning of traits versus rewards: dissociable neural correlates and effects on choice. Nat. Neurosci..

[18] Ramsey R, Ward R (2020). Challenges and opportunities for top-down modulation research in cognitive psychology. Acta Physiol. (Oxf).

[19] Sutherland CAM (2020). Individual differences in trust evaluations are shaped mostly by environments, not genes. Proc. Natl. Acad. Sci. USA.

[20] Sutherland CAM, Rhodes G, Burton NS, Young AW (2020). Do facial first impressions reflect a shared social reality?. Br. J. Psychol..

[21] Cook R, Over H (2020). A learning model can explain both shared and idiosyncratic first impressions from faces. Proc. Natl. Acad. Sci. USA.

[22] FeldmanHall O (2018). Stimulus generalization as a mechanism for learning to trust. Proc. Natl. Acad. Sci..

[23] Wang S, Falvello V, Porter J, Said CP, Todorov A (2018). Behavioral and neural adaptation in approach behavior. J. Cogn. Neurosci..

[24] Eggleston A, Flavell JC, Tipper SP, Cook R, Over H (2021). Culturally learned first impressions occur rapidly and automatically and emerge early in development. Dev. Sci..

[25] Todorov A (2017). Face Value: The Irresistible Influence of First Impressions.

[26] Over H, Eggleston A, Cook R (2020). Ritual and the origins of first impressions. Philos. Trans. R. Soc. B Biol. Sci..

[27] Ryan MP, Reese V, Wagner RF (2018). Dermatological depictions in animated movies. Br. J. Dermatol..

[28] Klein H, Shiffman KS (2006). Messages about physical attractiveness in animated cartoons. Body Image.

[29] Walle EA, Reschke PJ, Knothe JM (2017). Social referencing: defining and delineating a basic process of emotion. Emot. Rev..

[30] Weisbuch M, Pauker K, Ambady N (2009). The subtle transmission of race bias via televised nonverbal behavior. Science.

[31] Castelli L, Carraro L, Pavan G, Murelli E, Carraro A (2012). The power of the unsaid: the influence of nonverbal cues on implicit attitudes. J. Appl. Soc. Psychol..

[32] Skinner AL, Olson KR, Meltzoff AN (2019). Acquiring group bias: Observing other people’s nonverbal signals can create social group biases. J. Pers. Soc. Psychol..

[33] Repacholi BM, Meltzoff AN, Toub TS, Ruba AL (2016). Infants’ generalizations about other people’s emotions: foundations for trait-like attributions. Dev. Psychol..

[34] Shutts K, Banaji MR, Spelke ES (2010). Social categories guide young children’s preferences for novel objects. Dev. Sci..

[35] Cogsdill EJ, Banaji MR (2015). Face-trait inferences show robust child-adult agreement: Evidence from three types of faces. J. Exp. Soc. Psychol..

[36] Over H, Carpenter M (2015). Children infer affiliative and status relations from watching others imitate. Dev. Sci..

[37] Charlesworth TES, Hudson S-KTJ, Cogsdill EJ, Spelke ES, Banaji MR (2019). Developmental psychology children use targets’ facial appearance to guide and predict social behavior. Dev. Psychol..

[38] Dalrymple KA, Gomez J, Duchaine B (2013). The dartmouth database of children’s faces: acquisition and validation of a new face stimulus set. PLoS ONE.

[39] DeBruine, L. Webmorph (Version v0. 0.0. 9001). *Webmorph (Version v0.0.0.9001)* (2017).

[40] Tiddeman B, Burt M, Perrett D (2001). Prototyping and transforming facial textures for perception research. IEEE Comput. Graph. Appl..

[41] Tiddeman BP, Stirrat MR, Perrett DI (2005). Towards realism in facial image transformation: results of a wavelet MRF method. Comput. Graph. Forum.

[42] Langlois JH, Roggman LA (1990). Attractive faces are only average. Psychol. Sci..

[43] Little AC, Hancock PJB (2002). The role of masculinity and distinctiveness in judgments of human male facial attractiveness. Br. J. Psychol..

[44] Erdfelder E, Faul F, Buchner A, Lang AG (2009). Statistical power analyses using G*Power 3.1: tests for correlation and regression analyses. Behav. Res. Methods.

[45] Skinner AL, Meltzoff AN, Olson KR (2017). “Catching” social bias: Exposure to biased nonverbal signals creates social biases in preschool children. Psychol. Sci..

[46] Richter N, Tiddeman B, Haun DBM (2016). Social preference in preschoolers: effects of morphological self-similarity and familiarity. PLoS ONE.

[47] Brey E, Pauker K (2019). Teachers’ nonverbal behaviors influence children’s stereotypic beliefs. J. Exp. Child Psychol..

[48] Todorov A, Olivola CY, Dotsch R, Mende-Siedlecki P (2015). Social attributions from faces: determinants, consequences, accuracy, and functional significance. Ann. Rev. Psychol..

[49] Taylor MJ, Batty M, Itier RJ (2004). The faces of development: A review of early face processing over childhood. J. Cogn. Neurosci..

[50] Short LA, Lee K, Fu G, Mondloch CJ (2014). Category-specific face prototypes are emerging, but not yet mature, in 5-year-old children. J. Exp. Child Psychol..

[51] Germine L (2015). Individual aesthetic preferences for faces are shaped mostly by environments, not genes. Curr. Biol..

[52] Li Y, Jiao X, Liu Y, Tse C, Dong Y (2020). Age differences in facial trustworthiness judgement based on multiple facial cues. Br. J. Psychol..

[53] Olszanowski M, Parzuchowski M, Szymków A (2019). When the smile is not enough: the interactive role of smiling and facial characteristics in forming judgments about trustworthiness and dominance. Rocz. Psychol..

[54] Gao X, Maurer D (2009). Influence of intensity on children’s sensitivity to happy, sad, and fearful facial expressions. J. Exp. Child Psychol..

[55] DeBruine LM (2002). Facial resemblance enhances trust. Proc. R. Soc. B Biol. Sci..

[56] Efferson C, Vogt S (2013). Viewing men’s faces does not lead to accurate predictions of trustworthiness. Sci. Rep..

