# Author Correction: Young children learn first impressions of faces through social referencing

**DOI:** 10.1038/s41598-021-01762-w

**Published:** 2021-11-08

**Authors:** Adam Eggleston, Elena Geangu, Steven P. Tipper, Richard Cook, Harriet Over

**Affiliations:** 1 Department of Psychology, University of York, York, YO10 5DD UK; 2 Department of Psychological Sciences, University of London, London, UK

Correction to: *Scientific Reports* 10.1038/s41598-021-94204-6, published online 20 July 2021

The Acknowledgements section in the original version of this Article was incomplete.

“We would like to thank Bethany Fisher for assistance with coding and Dr Dalrymple and colleagues for sharing their stimuli with us.”

now reads:

“We thank Bethany Fisher for coding and Dr Dalrymple and colleagues for sharing their stimuli. This research was supported by the European Research Council under the European Union's Horizon 2020 Programme, grant number ERC-STG-755719 and a Philip Leverhulme Prize, both awarded to Harriet Over. Richard Cook was also supported by the European Research Council (ERC-2016-StG-715824).”

The original Article has been corrected.

