# Supplementary material for: Young children learn first impressions of faces through social referencing
Source: Sci Rep. 2021 Jul 20;11:14744. doi: 10.1038/s41598-021-94204-6 (PMC8292491; doi:10.1038/s41598-021-94204-6)
Supplement: Supplementary file 1 — Supplementary Information. [file 41598_2021_94204_MOESM1_ESM.pdf]

Supplementary Table S1

Table S1. Average (SD) trustworthiness/niceness ratings for targets by pairings.

Gender	Target A	Target B
Male	36.80 (20.44)	37.30 (22.96)
Male	31.45 (23.76)	35.10 (19.98)
Male	54.30 (20.62)	55.10 (19.00)
Male	57.10 (14.84)	59.60 (14.35)
Female	30.90 (20.23)	36.00 (20.50)
Female	42.00 (18.48)	48.90 (17.35)
Female	49.05 (19.45)	49.15 (17.45)
Female	55.55 (15.00)	57.8 (16.89)
